# Genetic variation in *UGT1A1* is not associated with altered liver biochemical parameters in healthy volunteers participating in bioequivalence trials

**DOI:** 10.3389/fphar.2024.1389968

**Published:** 2024-05-03

**Authors:** Eva González-Iglesias, Dolores Ochoa, Manuel Román, Paula Soria-Chacartegui, Samuel Martín-Vilchez, Marcos Navares-Gómez, Alejandro De Miguel, Pablo Zubiaur, Andrea Rodríguez-Lopez, Francisco Abad-Santos, Jesús Novalbos

**Affiliations:** ^1^ Clinical Pharmacology Department, Hospital Universitario de La Princesa, Instituto de Investigación Sanitaria La Princesa (IIS-Princesa), Faculty of Medicine, Universidad Autónoma de Madrid (UAM), Madrid, Spain; ^2^ Centro de Investigación Biomédica en Red de Enfermedades Hepáticas y Digestivas (CIBERehd), Instituto de Salud Carlos III, Madrid, Spain

**Keywords:** Bioequivalence trials, genetics, *UGT1A1* gene, Gilbert’s syndrome, liver enzymes

## Abstract

**Introduction:** Bioequivalence clinical trials are conducted in healthy volunteers whose blood tests should be within normal limits; individuals with Gilbert syndrome (GS) are excluded from these studies on suspicion of any liver disease, even if the change is clinically insignificant. GS is a benign genetic disorder characterized by elevated bilirubin levels, the primary cause of which is the presence of polymorphisms in *UGT1A1* gene. In this work, subjects with UGT1A1 intermediate (IM) or poor (PM) metabolizer genotype-informed phenotypes were investigated to determine whether they have a higher incidence of liver disease or other biochemical parameters.

**Methods:** The study population comprised 773 healthy volunteers who underwent biochemical analysis at baseline and at the end of the study which were genotyped for UGT1A1*80 (rs887829), as an indicator of UGT1A1*80+*28 (rs887829 and rs3064744), and UGT1A1*6 (rs4148323).

**Results:** Bilirubin levels were higher in subjects IMs and PMs compared to normal metabolizers (NMs). Decreased uric acid levels was observed in PMs compared to NMs. No associations were observed in liver enzyme levels according to UGT1A1 phenotype.

**Discussion:** Considering that there is no hepatic toxicity in subjects with UGT1A1 IM or PM phenotype, who are more likely to develop GS, this study suggests that they could be included in bioequivalence clinical trials as their biochemical parameters are not affected outside normal ranges.

## 1 Introduction

Bioequivalence clinical trials involve healthy volunteers, whose blood tests must be within the normal ranges, which is very stringent for bilirubin and liver enzymes. Gilbert’s syndrome (GS) is a benign genetic disorder related to the metabolism of bilirubin in liver (Düzenli et al., 2021). Bilirubin is the last product of heme catabolism that comes mostly from the breakdown of erythrocyte hemoglobin in the reticuloendothelial system (Memon et al., 2016). Bilirubin elimination is conducted by converting it into direct bilirubin by conjugation with glucuronic acid (Gil and Sąsiadek, 2012). Since patients with GS have reduced level of glucuronidation and unconjugated bilirubin is not water-soluble as conjugated bilirubin, it cannot be excreted in bile and patient suffers from unconjugated hyperbilirubinemia and mild intermittent icterus (Thoguluva Chandrasekar et al., 2022). In healthy people, the normal level of bilirubin ranges from 0.1 to 1.2 mg/dL. However, the levels in GS patients usually ranges from 1.2 to 5.3 mg/dL (Gil and Sąsiadek, 2012). Therefore, GS patients with elevated bilirubin levels are excluded from bioequivalence studies due to the suspicion of any liver disease, even if the change is clinically insignificant and despite that it is well known that there is no alteration in liver enzymes in patients with this syndrome (Moreno et al., 1984; Sidorenko et al., 2022; Vítek and Tiribelli, 2023).

GS patients carry variants in the gene encoding for the enzyme responsible of converting unconjugated bilirubin into conjugated bilirubin, the uridine diphosphoglucuronate-glucuronosyltransferase 1A1 (*UGT1A1*) (Thoguluva Chandrasekar et al., 2022). More specifically, it is related to the short tandem repeat (STR) variation in the promotor of this gene that consists in an addition of a dinucleotide sequence (TA) into the transcription initiation sequence A(TA)_7_TAA, converting it into A(TA)_8_TAA (Horsfall et al., 2011; Thoguluva Chandrasekar et al., 2022). This variation was called allele UGT1A1*28 (rs3064744) and it was previously annotated as rs34815109 or rs34983651 (Aronica et al., 2022). As a result, having this variant makes the enzyme have only 30% of the normal activity. In addition, this position in the genome also defines other alleles such as UGT1A1*36 when one dinucleotide sequence is deleted (A(TA)_6_TAA) or UGT1A1*37 when two TAs are added (A(TA)_9_TAA). The UGT1A1*36 appears to have higher transcript levels than UGT1A1*1, while UGT1A1*37 appears to have lower levels (Gammal et al., 2016). These variants are less common or may be absent depending on the geographic region of ancestry (Gammal et al., 2016).

Not every person that has the allele UGT1A1*28 finally develops visible symptoms as it depends on environmental factors like physical stress, prolonged fasting, poor diet, hemolytic reactions, febrile illnesses and menstruation (Düzenli et al., 2021). For example, a reduced caloric uptake to 400 kcal diary produces a 2 to 3-fold increase of bilirubin concentration in 48 h. GS usually appears during early adolescence and its more frequently diagnosed in males that females due to differences in sex steroids concentration and higher production of bilirubin in males (Thoguluva Chandrasekar et al., 2022).

The *UGT1A1* rs887829 C>T variant (UGT1A1*80) was studied for having a possible relation to UGT1A1*28. It has been described to be in almost complete linkage disequilibrium with UGT1A1*28 (r^2^ ≅ 0.99), although there are other variants in this STR that prevent complete disequilibrium (Gammal et al., 2016; Bravo-Gómez et al., 2022). It was reported that C allele was correlated with UGT1A1*1 and UGT1A1*36 and T allele with UGT1A1*28 and UGT1A1*37 (Gammal et al., 2016; Université Laval, 2022). Therefore, they describe that analysis of this allele could be used as an indicator of UGT1A1*80+*28 and thereby to infer the metabolic phenotype of UGT1A1, as it is a faster and more cost-effective genotyping technique than UGT1A1*28 genotyping (Bravo-Gómez et al., 2022).

Another allele was associated with the development of GS due to the decrease in enzyme activity such as UGT1A1*6 (rs4148323) (Han et al., 2006). It was identified as a predictor of bilirubin concentration in East Asian populations, where it accounts for about 5% of the variability (Gammal et al., 2016). Despite being worldwide widespread, differences depending on the ethnicity are found, being Sub-Saharan African the ones with highest prevalence (15%–25%), Europeans in the middle (5%–10%) and East Asian with the lowest prevalence (0%–5%) (Gil and Sąsiadek, 2012).

The main objective of this work was to investigate whether volunteers with UGT1A1 IM and PM phenotypes have a higher incidence of analytical changes in liver parameters. As a secondary objective, we sought to discover a possible relationship between UGT1A1 phenotype and the alteration of other biochemical parameters.

## 2 Materials and methods

### 2.1 Study population

The participants of this study were healthy volunteers enrolled in 29 bioequivalent trials of different drugs conducted at the Clinical Trials Unit of Hospital Universitario La Princesa (UECHUP), Madrid (Spain) (https://www.iis-princesa.org/infraestructuras/ensayos-clinicos/informacion-para-promotores/). The study drugs were: donepezil/memantine 10 mg/10 mg, cinitapride 1 mg, dabigatran 150 mg, dutasteride/tamsulosin 0.5 mg/0.4 mg, sitagliptin 100 mg, atorvastatin 80 mg, ezetimibe/atorvastatin 10 mg/80 mg, ibuprofen/chlorphenamine/phenylephrine 400 mg/2 mg/7.5 mg, dexketoprofen 25 mg, vildagliptin/metformin 50 mg/1,000 mg, olanzapine 2.5 mg and 5 mg, quetiapine 25 mg and 50 mg, rasagiline 1 mg, and eslicarbacepine 800 mg.

The European Medicines Agency (EMA) guideline for bioequivalence studies states that the subject population for bioequivalence studies should be selected with the aim of detecting differences between pharmaceutical products (European Medicines Agency, 2010). Therefore, these studies should normally be conducted in healthy volunteers who should meet inclusion/exclusion criteria clearly stated in the protocol. In the case of the studies included in this study, the inclusion criteria were: men or women aged from 18 to 55, free from organic or psychic conditions, with normal medical records, vital signs, electrocardiogram and physical examination and without significant abnormalities in hematology, coagulation, biochemistry, serology and urine analysis. The exclusion criteria were: having received medication 2 days prior to the start of the study, having a body mass index (BMI) outside the 18.5–30.0 range, being pregnant or breastfeeding women, having history of sensitivity to any drug, having a positive drug screening, smoking or alcoholism, blood donation in the last month and participation in another study with investigational drugs in the three previous months. In addition, the EMA also states that phenotyping and/or genotyping of subjects may be considered for safety or pharmacokinetic reasons.

All the clinical trials were approved by the Spanish Drugs Agency (AEMPS) and the Research Ethics Committee (CEIm) of the Hospital Universitario La Princesa. The development of the trials and the handling of data were conducted in compliance with Spanish Legislation and the International Council on Harmonization (ICH) guidelines on Good Clinical Practice (Vijayananthan and Nawawi, 2008). Volunteers signed an informed consent to participate in the study after being informed of the implications of their participation. During the development of the clinical trials, subjects were informed of the opportunity to participate in the pharmacogenetic study. The informed consent for the pharmacogenetic study (code SFC-FG-2020-1, IRB/Code: 4176) was evaluated by the IRB/EC board of Hospital Universitario La Princesa and approved on 9 July 2020. Finally, 773 gave written consent to participate. Volunteers self-reported their biogeographical origin and it was standardized as reported by Huddart *et al.* (Huddart et al., 2019).

### 2.2 Biochemical parameters determination

During the clinical trial screening visit, volunteers underwent a complete biochemical and hematological analysis, as well as a general urine and drug test. In this work, different biochemical parameters were selected for analysis, such as total bilirubin, because of its relation with GS; the levels of liver glutamic oxaloacetic transaminase (GOT), glutamic pyruvic transaminase (GPT), alkaline phosphatase (ALP) and gamma glutamyl transpeptidase (GGT), because they reflect liver toxicity; as well as other parameters not related to the liver, such as hemoglobin, uric acid and creatinine. In the same way, another blood sample was also obtained in the follow-up period of the clinical trial for the determination of the same biochemical parameters, which corresponds to 5–10 days after the last dosage of the study drug.

### 2.3 Genotyping and phenotyping

DNA from blood samples was extracted using a MagNA Pure instrument (Roche Applied Science, United States) or a Maxwell^®^ RSC Automated DNA extractor (Promega Biotech Iberica S.L). Genotyping of the two *UGT1A1* single nucleotide variants (SNVs) of interest (rs4148323 and rs887829) was carried out in a QuantStudio 12 K Flex qPCR instrument with an OpenArray thermal block (Applied Biosystems, Thermofisher, United States) as they were included in a custom array with more variants for other pharmacogenes related to transport and metabolism. Techniques were performed in the Pharmacogenetics Unit of the Clinical Pharmacology Department of the Hospital Universitario de La Princesa, Madrid, Spain.

UGT1A1 phenotype was inferred based on genotype and according to the Clinical Pharmacogenetics Implementation Consortium (CPIC) guidelines for *UGT1A1* and atazanavir prescribing, published in 2016 (Gammal et al., 2016). UGT1A1*6 was analyzed only in 320 subjects due to different array designs. UGT1A1*80 (rs887829) was used as a surrogate predictor of UGT1A1*80+*28 (rs3064744).

### 2.4 Statistical analysis

SPSS software (version 23, SPSS Inc., Chicago, IL, United States) was used to perform the statistical analysis. A chi-squared test was performed to examine the interaction between sex, phenotype, and biogeographic origin. The *p*-value used for statistical significance in this test was *p* < 0.05. Biochemical parameters were analyzed according to sex, biogeographical origin, UGT1A1 phenotype and the genotype for the two alleles separately, UGT1A1*6 and UGT1A1*80. Variable distributions were checked for normality with a Shapiro–Wilks test. All biochemical parameters followed a non-normal distribution, and therefore non-parametric tests were used. A Mann–Whitney test was used for variables with two categories and a Kruskal–Wallis test for those with three or more categories. Multivariate analysis was performed by linear regression, including those independent variables that were significantly associated with the dependent variable in the univariate analysis. Multivariate *p*-values (*p*_mv_), unstandardized β-coefficients (β) and R^2^ were shown for significant associations.

## 3 Results

### 3.1 Demographic characteristics

Study population was composed of 414 men (53.56%) and 359 women (46.44%), with a higher age in women than in men (29.40 ± 9.17 years old and 27.68 ± 7.35 years old, p univariate (puv) = 0.045). Distribution of healthy volunteers considering sex and phenotype *versus* biogeographic origin was shown in Table 1. Since only one volunteer was self-identified as American, one as Near Eastern, one as East Asian and six as Sub-Saharan African, they were merged into a single group under the name “Other”.

**TABLE 1:** Distribution of healthy volunteers considering sex and phenotype *versus* biogeographic origin.

Sex	Phenotype	Biogeographical origin			Total
		European	Latin-American	Other^#^	
Men	NM	141 (59.0%)	61 (36.1%)	1 (16.7%)	203 (49.0%)
	IM	86 (36.0%)	79 (46.7%)	5 (83.3%)	170 (41.1%)
	PM	12 (5.0%)	29 (17.2%)	0 (0.0%)	41 (9.9%)
Women	NM	84 (43.1%)	70 (43.5%)	0 (0.0%)	154 (42.9%)
	IM	80 (41.0%)	66 (41.0%)	2 (66.7%)	148 (41.2%)
	PM	31 (15.9%)	25 (15.5%)	1 (33.3%)	57 (15.9%)
Total		434	330	9	773

NM: normal metabolizer. IM: intermediate metabolizer. PM: poor metabolizer. # Other: one American, one Near Eastern, one East Asian, six Sub-Saharan African. Percentages calculated by sex.

Significant differences were observed in the distribution of phenotypes by sex in Europeans, Latin-Americans and overall (*p* < 0.001, *p =* 0.032 and *p =* 0.002, respectively) (Table 1). In Europeans, 62.7% of NMs and 51.8% of IMs were men, but 72.1% of PMs were women. Among Latin-Americans, 53.4% of NMs are women, while 54.5% of IMs and 53.7% of PMs are men. In the Other group, there is only one NM who is men and one PM who is women. As for the IMs, 71.4% are men. Significant differences in the distribution of phenotypes between Europeans and Latin-Americans was observed in men (*p* < 0.001), but not in women.

Out of 157 volunteers who did not present the *80 allele only two were *1/*6. The allele frequencies calculated by race showed that in our European population the frequency of *6 was 0% and that of *80 was 29.03%. For Latin Americans, the frequency of *6 was 1.67%, and that of *80 was 37.73%.

### 3.2 Biochemical parameters

In the screening analysis, all 8 biochemical parameters showed a lower value in women compared to men (*p*_uv_) < 0.001. These differences were also maintained in the multivariate analysis for total bilirubin (*p*mv < 0.001, β = −0.151, R^2^ = 0.137), hemoglobin (*p*mv < 0.001, β = −2.044, R^2^ = 0.563), uric acid (*p*mv < 0.001, β = −1,538, R^2^ = 0.386), creatinine (*p*mv < 0.001, β = −0.215, R^2^ = 0.514) and liver enzymes GOT (*p*mv < 0.001, β = −3.444, R^2^ = 0.065), GPT (*p*mv < 0.001, β = −6.324, R^2^ = 0.094), ALP (*p*mv < 0.001, β = −8.842, R^2^ = 0.099) and GGT (*p*_mv_<0.001, β = −5.748, R^2^ = 0.092) (Table 2).

**TABLE 2:** Biochemical parameters in screening according to sex, biogeographical origin, UGT1A1 phenotype and genotypes.

	N	BILT (mg/dL)	HGB (gr/dL)	URI (mg/dL)	CREA (mg/dL)	GOT (U/L)	GPT (U/L)	ALP (U/L)	GGT (U/L)
Sex									
Men	414	0.77 (0.37)	15.60 (0.91)	5.55 (1.09)	0.91 (0.11)	22.42 (7.31)	22.10 (11.43)	72.66 (19.82)	21.63 (14.49)
Women	359	0.63 (0.32)*	13.56 (0.88)*	4.00 (0.84)*	0.70 (0.10)*	18.97 (5.25)*	15.76 (7.26)*	64.13 (18.66)*	16.07 (8.85)*
Biogeographical origin									
European	434	0.71 (0.35)	14.69 (1.32)	4.91 (1.29)	0.83 (0.15)	20.70 (6.90)	18.92 (10.06)	64.74 (19.13)	16.79 (9.83)
Latin-American	330	0.70 (0.36)	14.60 (1.41)	4.70 (1.18)	0.78 (0.15)*^1^	20.75 (6.30)	19.29 (10.40)	74.02 (19.40)*^1^	22.06 (14.95)*^1^
Other^#^	9	0.66 (0.25)	14.80 (1.36)	5.52 (1.31)	0.90 (0.20)	25.78 (6.48)	24.78 (10.53)	64.44 (18.35)	17.89 (5.78)
UGT1A1 phenotype									
NM	357	0.59 (0.27)	14.71 (1.38)	4.97 (1.27)	0.82 (0.15)	20.83 (6.78)	18.96 (10.53)	68.22 (20.44)	18.98 (13.43)
IM	318	0.72 (0.29)*^2^	14.68 (1.35)	4.79 (1.24)	0.81 (0.15)	21.20 (7.13)	19.96 (10.86)	69.29 (18.72)	19.50 (12.45)
PM	98	1.10 (0.49)*^2^	14.34 (1.28)	4.46 (1.14)*^2^	0.80 (0.14)	19.48 (3.94)	17.17 (5.65)	68.47 (20.57)	17.85 (8.67)
UGT1A1*80 genotype in subjects without *6									
*1/*1	357	0.59 (0.27)	14.71 (1.38)	4.97 (1.27)	0.82 (0.15)	20.83 (6.78)	18.96 (10.53)	68.22 (20.44)	18.98 (13.43)
*1/*80	316	0.71 (0.29)*^3^	14.68 (1.35)	4.78 (1.24)	0.81 (0.15)	21.25 (7.13)	20.01 (10.88)	69.23 (18.79)	19.47 (12.41)
*80/*80	96	1.11 (0.49)*^3^	14.37 (1.28)	4.47 (1.15)*^3^	0.80 (0.14)	19.54 (3.92)	17.29 (5.64)	68.26 (20.71)	17.95 (8.72)
UGT1A1*6 genotype in subjects without *80									
*1/*1	155	0.56 (0.23)	14.90 (1.36)	5.03 (1.26)	0.83 (0.15)	21.25 (7.28)	20.23 (11.45)	70.40 (20.42)	18.98 (16.06)
*1/*6	2	0.80 (0.16)	15.70 (0.99)	5.10 (0.85)	0.90 (0.00)	14.50 (0.71)	11.00 (1.41)	79.00 (4.24)	25.50 (23.33)

Data are shown as mean (standard deviation) although these are non-parametric tests following the central limit theorem. BILT: total bilirubin. HGB: hemoglobin. URI: uric acid. CREA: creatinine. GOT: glutamic oxaloacetic transaminase. GPT: glutamic pyruvic transaminase. ALP: alkaline phosphatase. GGT: gamma glutamyl transpeptidase. # Other: American, Near Eastern, East Asian and Sub-Saharan African. * *univariate p-value* (*p*_uv_) < 0.05 compared to men. *^1^ *p*_uv_<0.05 compared to European. *^2^ *p*_uv_<0.05 compared to NM. *^3^ *p*_uv_<0.05 compared to *1/*1. underlined: *multivariate p-value* (*p*_mv_) < 0.05.

A lower value in creatinine levels was observed in Latin-Americans in comparison to Europeans (*p*uv < 0.001, *p*mv < 0.001, β = −0.038, R^2^ = 0.514). Moreover, higher ALP and GGT levels were found in Latin-Americans compared to Europeans (*p*uv < 0.001, *p*mv < 0.001, β = 9.331, R^2^ = 0.099; *p*uv < 0.001, *p*mv < 0.001, β = 5.363, R^2^ = 0.092, respectively) (Table 2).

Of the 773 subjects enrolled, 50 had out-of-range bilirubin levels (range 1.3–3 mg/dl) as an exception because no other subjects were available who met the inclusion criteria. Of these, 7 were NMs (1.96%), 16 IMs (5.03%) and 27 PMs (27.55%). A significantly higher total bilirubin level was observed in UGT1A1 PMs and IMs compared to NMs, with significant differences between IMs and PMs (*p*uv < 0.001, *p*mv < 0.001, β = 0.112, R^2^ = 0.137). Finally, a lower uric acid level was also observed in PMs compared to NMs (*p*_uv_ = 0.001, *p*_mv_ = 0.020, β = −0.082, R^2^ = 0.386). No associations were found between the UGT1A1 phenotype and changes in liver enzyme levels or other biochemical parameters. No alterations in any serum parameters outside the normal range were observed (Table 2).

For UGT1A1*80, bilirubin levels were higher in *80/*80 and *1/*80 compared with *1/*1 (*p*uv < 0.001). The results also showed a lower uric acid level in *80/*80 *versus* *1/*1 (*p*_uv_ = 0.001). Due to the similarity of UGT1A1 phenotype and genotype results for UGT1A1*80, only the inferred phenotype was included in the multivariate analysis (Table 2). Finally, only two subjects had the *1/*6 genotype in absence of *80 allele; although the mean total bilirubin in these subjects was higher than the *1/*1, significant differences could not be found (*p*_uv_ = 0.143) (Table 2).

In the follow-up analysis, biochemical parameters were also statistically lower in women than in men (*p*_uv_ <0.001), also maintained in the multivariate analysis (Table 3). A lower value in uric acid and creatinine levels was observed in Latin-Americans in comparison to Europeans (*p*_uv_ = 0.041 and *p*_uv_ = 0.016, *p*_mv_ = 0.009, β = −0.022, R^2^ = 0.443, respectively). The group “Other” shows higher GOT level compared to Europeans and Latin-Americans (*p*_uv_ <0.001). The UGT1A1 phenotype showed the same effect in the follow up as in the screening for total bilirubin and uric acid. A lower hemoglobin value was observed in PMs compared to NMs and IMs (*p*_uv_ = 0.011 and *p*_uv_ = 0.032, respectively), that was not observed in the screening analysis. At the end of the study, 5 NMs had bilirubin levels above the range (1.40%), 11 IMs (3.46%) and 23 PMs (23.47%); 17 of them had bilirubin out of range at screening. The maximum level achieved during follow-up was 2.8 mg/dL for PMs, 1.8 mg/dL for IMs and 1.6 mg/dL for NMs. No changes were found in any of the other analytical parameters.

**TABLE 3:** Biochemical parameters in follow-up according to sex, biogeographical origin and UGT1A1 phenotype.

	N	BILT (mg/dL)	HGB (gr/dL)	URI (mg/dL)	CREA (mg/dL)	GOT (U/L)	GPT (U/L)	ALP (U/L)	GGT (U/L)
Sex									
Men	414	0.72 (0.35)	15.10 (1.02)	5.38 (1.01)	0.90 (0.12)	21.95 (9.42)	20.78 (10.77)	70.94 (19.39)	19.47 (13.66)
Women	359	0.58 (0.28)*	12.86 (1.01)*	3.95 (0.91)*	0.70 (0.11)*	18.68 (6.75)*	15.04 (6.40)*	63.76 (18.76)*	14.63 (8.50)*
Biogeographical origin									
European	434	0.65 (0.32)	14.15 (1.47)	4.81 (1.22)	0.82 (0.15)	20.24 (8.24)	17.67 (8.07)	63.44 (18.23)	15.07 (9.88)
Latin-American	330	0.65 (0.34)	13.94 (1.56)	4.58 (1.15)*^1^	0.79 (0.16)*^1^	20.36 (8.27)	18.51 (10.88)	73.06 (19.54)*^1^	20.04 (13.52)*^1^
Other^#^	9	0.63 (0.36)	14.26 (1.53)	5.29 (1.50)	0.87 (0.20)	32.22 (15.01)*^2^	24.78 (12.56)	68.44 (23.28)	17.22 (6.16)
UGT1A1 phenotype									
NM	357	0.56 (0.23)	14.14 (1.56)	4.88 (1.23)	0.81 (0.15)	20.39 (8.56)	18.04 (9.21)	66.64 (20.15)	17.62 (13.62)
IM	318	0.64 (0.27)*^3^	14.09 (1.47)	4.65 (1.12)	0.81 (0.16)	20.63 (7.94)	18.30 (8.24)	68.64 (18.47)	16.98 (10.58)
PM	98	1.03 (0.49)*^3^	13.64 (1.42)*^4^	4.32 (1.20)*^5^	0.79 (0.14)	19.95 (9.58)	17.73 (13.32)	67.74 (19.78)	16.54 (7.68)

Data are shown as mean (standard deviation) although these are non-parametric tests following the central limit theorem. BILT: total bilirubin. HGB: hemoglobin. URI: uric acid. CREA: creatinine. GOT: glutamic oxaloacetic transaminase. GPT: glutamic pyruvic transaminase. ALP: alkaline phosphatase. GGT: gamma glutamyl transpeptidase. # Other: American, Near Eastern, East Asian and Sub-Saharan African.* *univariate p-value* (*p*_uv_) < 0.05 compared to men. *^1^ *p*_uv_<0.05 compared to European. *^2^ *p*_uv_<0.05 compared to European and *p*_uv_<0.05 compared to Latin-American. *^3^ *p*_uv_<0.05 compared to NM. *^4^ *p*_uv_<0.05 compared to NM, and IM. *^5^ *p*_uv_<0.05 compared to NM.,

*p*_uv_<0.05 compared to NM., underlined: *multivariate p-value* (*p*_mv_) < 0.5.

## 4 Discussion

GS is a disorder of bilirubin metabolism that does not pose a health problem to the individual; in fact, its accumulation appears to have an antioxidant effect that may be protective. For this reason, GS has been associated with a lower prevalence of chronic diseases such as cardiovascular disease or type 2 diabetes, a lower incidence of ischemic heart disease, and a lower incidence of Hodgkin’s lymphoma and endometrial cancer compared to the general population (Wagner et al., 2018). However, the deficient hepatic metabolism of GS patients is an exclusion factor in clinical trials. This is due to the suspicion that other hepatic factors besides bilirubin metabolism may be affected, thus altering the results of bioequivalence clinical trials. For example, increased toxicity of the drug irinotecan has been described in patients with a PM phenotype for UGT1A1, such as those with GS (Lankisch et al., 2008).

CPIC, in its guideline on *UGT1A1* and atazanavir published in 2016, refers to a frequency of 0.79% for UGT1A1*6 and 31.42% for UGT1A1*80 for Europeans. Similarly, for Latin-Americans it refers a frequency of 1.16% for UGT1A1*6 and 38.27% for UGT1A1*80. The frequencies of UGT1A1*6 and UGT1A1*80 reflected in this study are very similar to CPIC (Gammal et al., 2016). The allele frequency of UGT1A1*6 reflected in the European population in our study is consistent with the frequency of this allele previously described in Spanish population (Cerezo-Arias et al., 2022). This study reflects the largest population genotyped for UGT1A1*80 to date.

Results of this study confirmed the existing sex differences in biochemical parameters that were already described in previous articles (Werner et al., 1970; Dufour et al., 2005; Rushton and Barth, 2010; O Leary et al., 2017). This is the case for total bilirubin, uric acid and ALP and GOT enzymes, for which was described the existence of higher serum levels of these parameters in males *versus* females after puberty possibly due to differences in sex hormone activity throughout life (Werner et al., 1970). Reference ranges for hemoglobin in women of reproductive age showed lower values than those of men of equivalent age; when in the absence of different biological needs, similar values would be expected. This may be because these ranges were obtained from large population-based studies that possibly included a significant number of women who spend a part of their lives iron deficient due to blood loss during menstruation (Rushton and Barth, 2010). As for creatinine, it was also known that women have lower serum creatinine values than men even though they have similar renal function, because men have greater muscle mass (O Leary et al., 2017). Finally, it was described that the reference ranges for liver enzymes GPT and GGT should be higher for men than for women, which corresponds to the differences found in this study (Dufour et al., 2005).

The existence of differences in the levels of some biochemical parameters depending on the biogeographic origin highlights the necessity of establishing specific reference intervals for each of them (Lim et al., 2015). Although the groups reflected in these articles are different from those included in this study, genetic variation between origins is proposed as the cause of these differences (Jones et al., 1998; DeBoer et al., 2012; Schneider et al., 2014; Beydoun et al., 2018; Mariño-Ramírez et al., 2022).

Our results confirmed that UGT1A1 phenotype allows differences in total bilirubin levels to be seen, showing a higher bilirubin in IMs, even higher in PMs (Lin et al., 2006; Chen et al., 2011; Bravo-Gómez et al., 2022). The small number of subjects who were UGT1A1*6 carriers may cause the results to show no significant increase in bilirubin levels, although it was shown to affect its disposition in previous researches (Barbarino et al., 2014; Gammal et al., 2016; Hanafusa et al., 2022).

To our knowledge, the *UGT1A1* gene has not been described to be involved in hemoglobin metabolism. The level in the PM is not outside the normal range. Moreover, all phenotypes decreased their hemoglobin levels at follow-up which can be explained by the blood draws performed during the clinical trial. The difference of PM was not maintained in multivariate analysis, suggesting that it could be a false positive. For all these reasons, we could suspect that this is a low value due to the assay itself.

Additionally, a lower uric acid level in subjects with the PM phenotype for UGT1A1 was observed compared to NM. Information on a possible relationship between the *UGT1A1* gene and plasma uric acid levels is scarce. Only one article mentions that patients with GS did not present alterations in uric acid levels, which is consistent with the results found in this article, since although slight differences are observed depending on the phenotype, in no case do they exceed the range of normality (Bulmer et al., 2008). More studies are needed to clarify whether there is a relationship between the two.

No difference in liver enzyme levels was found as a function of UGT1A1 phenotype. Previous articles examining GOT, GPT and GGT liver enzyme levels in GS patients also found the same results (Bulmer et al., 2008; Hsu et al., 2022). These data suggest that there was no underlying liver pathology causing elevated bilirubin levels, but that it was a consequence of the UGT1A1 phenotype. Therefore, we suggest that genotyping of this gene should be performed in those subjects with out-of-range bilirubin levels. NM individuals should be excluded because of suspected liver involvement due to other causes, but IM and PM volunteers could be included because their bilirubin levels are only due to the phenotype of the gene. It should also be considered that the phenotype of other genes that could alter the metabolism of the study drugs are not considered an exclusion criterion, e.g., PM for CYP2C19 or ultrarrapid metabolizers (UMs) for CYP2D6, but UGT1A1 as a cause of Gilbert’s syndrome is considered even though it is a benign disease. In today’s world we should try to include as much variability as possible to cover all individuals in society.

## 5 Limitations

This study has two major limitations. First, as this was a clinical trial with inclusion and exclusion criteria, subjects with high bilirubin levels were excluded. Therefore, most subjects with genotype corresponding to GS could have been excluded, reducing the prevalence of GS and possible reducing the existing differences both in total bilirubin levels and in other parameters studied. Second, because not all subjects were genotyped for the UGT1A1*6 allele, statistical power was reduced for evaluation of the relevance of this allele.

## 6 Conclusion

This study reflects the largest population genotyped for UGT1A1*80 to date. Bilirubin levels were higher in volunteers with IM and PM phenotypes for UGT1A1. Given that there is no hepatic involvement in these subjects who are more likely to develop GS, this study suggests that there is no underlying liver pathology causing elevated bilirubin levels, but that it is a consequence of the UGT1A1 phenotype. Therefore, IM and PM individuals could be included in clinical trials because their bilirubin levels are only due to the gene phenotype and not to other pathologies.

## Data Availability

The original contributions presented in the study are publicly available. This data can be found here: public repository of the Community of Madrid, https://hdl.handle.net/20.500.12530/87827.
